# *B-RAF* and *N-RAS* Mutations Are Preserved during Short Time In Vitro Propagation and Differentially Impact Prognosis

**DOI:** 10.1371/journal.pone.0000236

**Published:** 2007-02-21

**Authors:** Selma Ugurel, Ranjit K. Thirumaran, Sandra Bloethner, Andreas Gast, Antje Sucker, Jan Mueller-Berghaus, Werner Rittgen, Kari Hemminki, Jürgen C. Becker, Rajiv Kumar, Dirk Schadendorf

**Affiliations:** 1 Skin Cancer Unit, German Cancer Research Center Heidelberg and Department of Dermatology, University Medical Center Mannheim, Mannheim, Germany; 2 Division of Molecular Genetic Epidemiology, German Cancer Research Center, Heidelberg, Germany; 3 Central Unit of Biostatistics, German Cancer Research Center, Heidelberg, Germany; 4 Department of Biosciences, Karolinska Institute, Huddinge, Sweden; 5 Department of Dermatology, University of Wuerzburg, Germany; Ordway Research Institute, Inc., United States of America

## Abstract

In melanoma, the RAS/RAF/MEK/ERK signalling pathway is an area of great interest, because it regulates tumor cell proliferation and survival. A varying mutation rate has been reported for B-RAF and N-RAS, which has been largely attributed to the differential source of tumor DNA analyzed, e.g., fixed tumor tissues or in vitro propagated melanoma cells. Notably, this variation also interfered with interpreting the impact of these mutations on the clinical course of the disease. Consequently, we investigated the mutational profile of B-RAF and N-RAS in biopsies and corresponding cell lines from metastatic tumor lesions of 109 melanoma patients (AJCC stage III/IV), and its respective impact on survival. 97 tissue biopsies and 105 biopsy-derived cell lines were screened for B-RAF and N-RAS mutations by PCR single strand conformation polymorphism and DNA sequencing. Mutations were correlated with patient survival data obtained within a median follow-up time of 31 months. B-RAF mutations were detected in 55% tissues and 51% cell lines, N-RAS mutations in 23% tissues and 25% cell lines, respectively. There was strong concordance between the mutational status of tissues and corresponding cell lines, showing a differing status for B-RAF in only 5% and N-RAS in only 6%, respectively. Patients with tumors carrying mutated B-RAF showed an impaired median survival (8.0 versus 11.8 months, p = 0.055, tissues; 7.1 versus 9.3 months, p = 0.068, cell lines), whereas patients with N-RAS-mutated tumors presented with a favorable prognosis (median survival 12.5 versus 7.9 months, p = 0.084, tissues; 15.4 versus 6.8 months, p = 0.0008, cell lines), each in comparison with wildtype gene status. Multivariate analysis qualified N-RAS (p = 0.006) but not B-RAF mutation status as an independent prognostic factor of overall survival. Our findings demonstrate that B-RAF and N-RAS mutations are well preserved during short term in vitro propagation and, most importantly, differentially impact the outcome of melanoma patients.

## Introduction

Malignant melanoma is associated with genetic heterogeneity and a complex etiology. In contrast to other skin cancers, melanoma affects a younger population and has a strong tendency to metastasize with a consequently extremely poor overall survival. In the majority of melanomas, the RAS/RAF/MEK/ERK signalling pathway is constitutively activated either due to oncogenic mutations in B-RAF and N-RAS genes or through autocrine growth factor stimulation [1]. RAS proteins are membrane-bound small G proteins, whereas RAF, MEK, and ERK are cytosolic protein kinases that form a tiered protein kinase cascade downstream of RAS. Signalling is initiated, when active RAS recruits RAF to the plasma membrane for activation through a complex process requiring lipid and protein binding, conformational changes, and regulatory phosphorylation and dephosphorylation events. There are three RAF proteins in mammals, A-RAF, B-RAF, and C-RAF, which can all activate MEK, but clearly perform distinct functions in vivo as shown by the phenotypic differences between A-RAF, B-RAF, and C-RAF knock-out mice.

It has long been known that activating N-RAS codon 61 mutations occur in up to 30% of all cutaneous melanoma cases. In 2002, Davies et al. reported that B-RAF mutations occur at a high frequency in melanoma; mutations were found in 20 of 34 melanoma cell lines (59%), 12 of 15 short-term cultures (80%), and six of nine melanoma tumours (67%) [2]. Mutations in the B-RAF gene are mainly localized in the kinase activation domain with the majority involving the substitution of valine by acidic or basic residues at codon 600 [2], [3]. This mutation results in a strong activation of B-RAF, constitutively stimulating the MEK-ERK signaling pathway. In contrast, A-RAF and C-RAF have not been found to be mutated because their regulation is fundamentally different from that of B-RAF. The report by Davies et al. stimulated a large research effort, which confirmed the originally reported high frequency of B-RAF mutations in melanoma. However, this frequency ranges between 30% to 70%, a variation, that has been largely attributed to the differential source of the analysed tumor DNA, e.g. fixed tumor tissues or in vitro propagated melanoma cells. Notably, the variation also interfered with the conclusive interpretation of the impact of these mutations on the clinical course of the disease.

The observation that mutations in B-RAF and N-RAS are mutually exclusive lead to the hypothesis, that the activation of either B-RAF or N-RAS results in a similar cellular phenotype [3], [4], [5], [6], [7]. However, recent reports about the association of B-RAF and/or N-RAS mutations with the prognosis of melanoma patients revealed contradictory results [5], [7], [8], [9], [10], requiring clarification by further studies, particularly given the varying frequencies of the reported B-RAF and N-RAS mutations. The present study is the first to investigate the mutational profile of B-RAF and N-RAS in both, tumor tissue biopsies and corresponding, biopsy-derived cell lines from metastatic melanoma patients, in correlation with a putative impact on survival. The analysis included 97 tumor tissues and 105 cell lines from 109 melanoma patients with a median follow-up time of 31 months. The study is reported following the newly established REMARK guidelines [11].

## Methods

### Patients

Patients were enrolled in accordance with the following eligibility criteria: histologically confirmed melanoma of the skin, mucosa, or unknown primary; stage III or IV disease according to AJCC [12]; and at least one metastatic lesion accessible for a bioptic procedure. Patient inclusion was allowed with or without current systemic treatment. Patients with primary ocular melanomas were excluded. After a patient's written informed consent, one biopsy was obtained from either a solid metastatic lesions or a malignant effusion. Biopsies from solid lesions were subsequently divided into three parts: one was immediately frozen down in liquid nitrogen until further analysis, the second was used for histopathological confirmation of melanoma, and the third was used for establishing a permanently growing melanoma cell line. Patient charts were reviewed for characteristics of the primary tumor (site and histological type of primary). The patient's age and disease stage at the time of biopsy were recorded. Follow-up examinations were performed at least once every three months. All tumor samples and clinical data were collected with Institutional Review Board approval and patient's informed consent.

### Tissues and cell lines

The frozen solid tumor tissue samples were used for DNA isolation by RNase and proteinase K digestion and subsequent phenol-chloroform extraction [13]. The biopsy-derived cell lines were maintained in RPMI 1640 (Life Technologies, Grand Island, NY) supplemented with 10% fetal calf serum (FCS; Life Technologies), 5 mM L-glutamine, 100 U/ml penicillin and 100 µg/ml streptomycin at 37°C in a humidified 5% CO_2_ atmosphere. They were used for analysis not before six to eight culture passages. After growing until 70 to 80% confluence, the cells were gently detached using 0.05% ethylenediaminetetraacetic acid (EDTA)/phosphate-buffered saline (PBS), washed twice, resuspended in 10% FCS/RPMI and frozen down in liquid nitrogen. DNA was extracted using the Puregene DNA purification kit (Gentra Systems, Minneapolis, MN).

### Mutation detection by single strand conformation polymorphism (SSCP)

Fluorescent capillary SSCP technique was used to detect mutations in exon 15 of the B-RAF gene and exon 2 of the N-RAS gene. Briefly, the exons were amplified by PCR using primers labeled with 6-FAM and HEX fluorescent dye (Applied Biosystems, Foster City, CA) under conditions described earlier [6], [10]. The electrophoresis of the amplified products was carried out under non-denaturing conditions in a 16-array capillary sequencer (ABI3100; Applied Biosystems). Mutations were detected by differential migration patterns compared to fragments that contained wild type sequences. The analysis of the results was carried out using the GeneScan software (Applied Biosystems). In exon 11 of the B-RAF gene and in exon 1 of the N-RAS gene mutations were screened by ‘radio-active’ SSCP technique. The DNA fragments were amplified by PCR in presence of [α-^32^P] dCTP and the amplified products were electrophoresed on non-denaturing MDE gels under different conditions as described earlier [6], [10].

### Direct DNA sequencing

Direct DNA sequencing was used to identify and confirm mutations detected in the B-RAF and N-RAS genes by SSCP. For sequencing, PCR products were incubated with ExoSapIT (USB Amersham, Uppsala, Sweden) at 37°C for 30 min followed by heating to 85°C for 15 min. The sequencing reactions were carried out using the BigDye Terminator Cyle sequencing kit (Applied Biosystems) in a 10 µl volume containing purified PCR product and a sequencing primer. The temperature conditions set for sequencing reactions were 96^o^C for 2 minutes followed by 27 cycles at 96°C for 30 seconds, 54°C for 10 seconds and 60°C for 4 minutes. The reaction products were precipitated with 2-propanol, washed with 75% ethanol, resuspended in 25 µl water and loaded onto ABI prism 3100 Genetic Analyzer (Applied Biosystems). Both, forward and reverse strands were sequenced separately. Primary sequencing data were analyzed using a sequence analysis software (Sequence Analysis 3.7; Applied Biosystems) and comparative analysis was done with the online MultAlin software (http://www.prodes.toulouse.inra.fr/multalin/ multalin.html).

### Statistics

Survival curves and median survival times were, if not otherwise indicated, calculated from the date of biopsy until either death from melanoma or last patient contact, respectively, and are graphically presented using the Kaplan-Meier method for censored failure time data. The log rank test was used for comparing survival probabilities. The multivariate proportional hazards regression of Cox was used to assess the impact of multiple prognostic factors on survival. The factors tested were mutational status of B-RAF and N-RAS, gender, disease stage at biopsy, and site of primary tumor. Statistical analyses were performed using the statistical packages ADAM (Central Unit for Biostatistics, German Cancer Research Center Heidelberg, Germany) and SAS 8.1 (SAS Institute, Cary, NC). Differences with a p value <0.05 were considered statistically significant.

## Results

**Figure 1:**
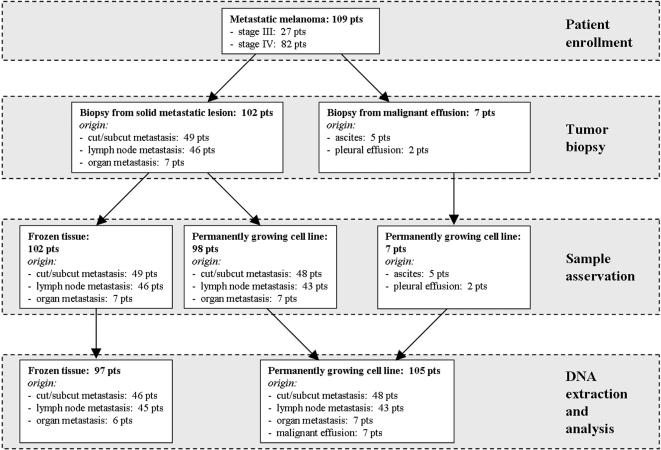
Schematic presentation of the study flow.

**Figure 2:**
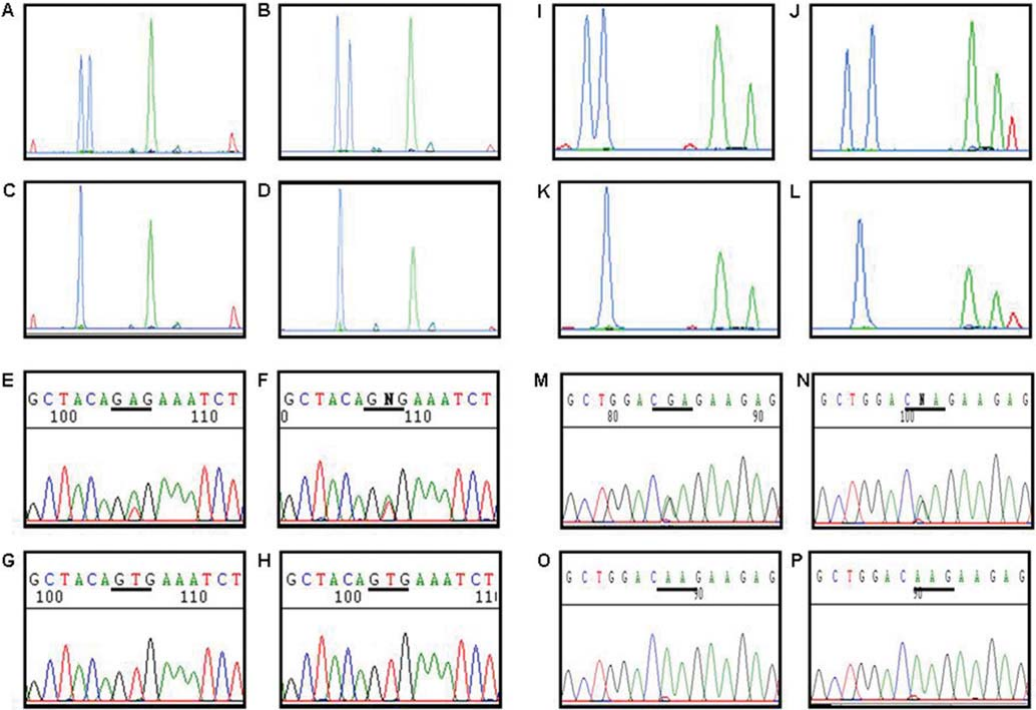
Detection and identification of B-RAF and N-RAS mutations in melanoma cell lines and corresponding tissues by fluorescent capillary electrophoresis SSCP and DNA sequencing. Migration patterns under non-denaturing conditions of single stranded fragments of exon 15 of the B-RAF gene with a T1799A mutation at codon 600 in the cell line Ma-Mel-36 (A) and the corresponding tumor tissue (B). Migration patterns for the B-RAF exon 15 fragments with wild type sequences in the cell line Ma-Mel-37a (C) and the corresponding tumor tissue (D). Panels (E) to (H) show sequence analyses of the cell lines and tumor tissues given in (A) to (D). Fluorescent capillary electrophoresis patterns for the N-RAS exon 2 sequence with a CAA>CGA mutation at codon 61 in the cell line Ma-Mel-05 (I) and the corresponding tumor tissue (J). Migration patterns for the N-RAS exon 2 fragments with wild-type sequences in the cell line Ma-Mel-59 (K) and the corresponding tumor tissue (L). Panels (M) to (P) show the confirmation of the mutations in the cell lines and tissues shown in (I) to (L) by sequence analysis.

109 metastatic melanoma patients were enrolled into the study (***Table 1***); the patient flow is presented in ***Figure 1***. The median follow-up time was 31 months. Biopsies were obtained from 102 solid metastatic lesions and seven malignant effusions. The solid lesions included 48% cutaneous or subcutaneous metastases, 45% lymph node metastases, and 7% organ metastases (brain, liver, lung, small bowel, urinary bladder and kidney). Routine histopathology confirmed metastases from melanoma in all cases. The malignant effusions originated from ascites (five patients) and pleura (two patients). Permanently growing melanoma cell lines could be established from 98 out of 102 solid lesions and from all seven effusions. DNA of analysis grade could be isolated from 97 out of 102 tissue biopsies and from all 105 biopsy-derived cell lines, and screened for mutations in exons 11 and 15 of the B-RAF gene and exons 1 and 2 of the N-RAS gene. Detailed patient characteristics as well as mutational profiles of tumor tissues and cell lines are presented in ***Table 2***. Representative data from SSCP analysis and DNA sequencing are shown in ***Figure 2***.

**Table 1:** Patient characteristics.

Patients enrolled		109 (100.0%)
**Gender**	male	59 (54.1%)
	female	50 (45.9%)
**Age at biopsy ** ***(years)***	median (range)	56 (14–87)
**Stage at biopsy ** ***(AJCC)***	III	27 (24.8%)
	IV	82 (75.2%)
**Site of primary**	skin	84 (77.1%)
	mucosa	2 (1.8%)
	occult	14 (12.8%)
	n.a.	9 (8.3%)
**Type of primary**	NM	30 (27.6%)
	SSM	20 (18.3%)
	ALM	7 (6.4%)
	LMM	2 (1.8%)
	mucosa	2 (1.8%)
	amelanotic	2 (1.8%)
	occult	14 (12.9%)
	n.a.	32 (29.4%)

Patients were enrolled in accordance with the following eligibility criteria: histologically confirmed melanoma of the skin, mucosa, or unknown primary; stage III or IV disease; and at least one metastatic lesion accessible for a bioptic procedure. AJCC, American Joint Committee on Cancer; NM, nodular melanoma; SSM, superficial spreading melanoma; ALM, acrolentiginous melanoma; LMM, lentigo maligna melanoma; occult, melanoma of unknown primary; n.a., not available.

**Table 2:** Detailed patient characteristics and tumor mutation status.

Patient ID	Age at biopsy (years)	Gender	Localization of primary	Type of primary	Stage at biopsy (AJCC)	Biopsy origin	B-Raf status in tissue biopsy	N-Ras status in tissue biopsy	Cell line established from biopsy	B-Raf status in cell line	N-Ras status in cell line	Survival after biopsy (months)
1	57	m	n.a.	n.a.	IV	LN	G469V	wt	n.a.	n.d.	n.d.	4.0
2	36	f	skin	n.a.	IV	pleural effusion	n.d.	n.d.	UKRV-Mel-02	V600E	wt	0.5
3	33	f	n.a.	n.a.	IV	C/SQ	V600E	wt	UKRV-Mel-03	V600E	wt	7.0
4	51	f	skin	NM	IV	C/SQ	V600E	wt	UKRV-Mel-06b	V600E	wt	4.1
5	82	m	n.a.	n.a.	IV	LN	V600E	wt	UKRV-Mel-11	wt	wt	12.0
6	29	m	n.a.	n.a.	IV	C/SQ	V600E	wt	UKRV-Mel-12	V600E	wt	8.0
7	52	f	skin	SSM	IV	LN	V600E	wt	UKRV-Mel-14b	V600E	wt	13.0
8	48	f	skin	NM	IV	LN	n.d.	n.d.	UKRV-Mel-15	wt	Q61R	15.4
9	24	m	n.a.	n.a.	IV	C/SQ	V600E	wt	UKRV-Mel-16	V600E	wt	6.0
10	48	m	skin	n.a.	IV	LN	V600E	wt	UKRV-Mel-17	V600E	wt	5.0+
11	53	f	n.a.	n.a.	IV	LN	V600E	wt	UKRV-Mel-19	V600E	wt	6.0
12	27	m	skin	n.a.	IV	C/SQ	V600E	wt	UKRV-Mel-20a	V600E	wt	9.8
13	40	m	n.a.	n.a.	IV	LN	V600E	wt	UKRV-Mel-24	V600E	wt	9.0
14	39	m	n.a.	n.a.	IV	C/SQ	wt	wt	UKRV-Mel-29	V600K	wt	12.0
15	58	m	skin	n.a.	IV	C/SQ	V600E	wt	UKRV-Mel-31	V600E	wt	3.1
16	78	m	skin	LMM	IV	C/SQ	wt	Q61R	Ma-Mel-02	wt	wt	6.0
17	29	m	skin	n.a.	IV	urinary bladder	n.d.	n.d.	Ma-Mel-04	V600E	wt	13.0
18	49	f	skin	n.a.	IV	C/SQ	wt	Q61R	Ma-Mel-05	wt	Q61R	1.9
19	58	m	skin	NM	IV	LN	V600E	wt	Ma-Mel-06	V600E	wt	0.3+
20	68	m	skin	NM	IV	LN	V600E	wt	Ma-Mel-07	V600E	wt	11.8
21	56	m	skin	NM	III	C/SQ	wt	wt	Ma-Mel-08a	wt	wt	47.1
22	50	m	skin	SSM	IV	C/SQ	V600E	wt	Ma-Mel-13	V600E	wt	37.7
23	64	f	skin	NM	IV	C/SQ	V600E	wt	Ma-Mel-14	V600E	wt	14.2
24	39	f	occult	occult	IV	LN	wt	wt	Ma-Mel-15	wt	wt	1.0+
25	31	m	skin	amelanotic	IV	small bowel	V600E	wt	Ma-Mel-16	V600E	wt	2.9+
26	62	f	skin	SSM	IV	C/SQ	V600E	wt	Ma-Mel-19	V600E	wt	0.2+
27	56	f	skin	NM	IV	C/SQ	V600E	wt	Ma-Mel-20	V600E	wt	1.2
28	78	f	skin	n.a.	IV	kidney	wt	Q61R	Ma-Mel-22	wt	Q61R	42.7
29	44	f	skin	SSM	IV	C/SQ	n.d.	n.d.	Ma-Mel-25	wt	Q61K	33.6
30	73	m	skin	n.a.	III	LN	wt	Q61R	Ma-Mel-26a	wt	Q61R	6.3
31	58	f	skin	ALM	IV	C/SQ	wt	wt	Ma-Mel-27	wt	G12D	7.5
32	65	f	occult	occult	IV	C/SQ	wt	Q61R	Ma-Mel-28	wt	Q61R	4.6
33	58	m	skin	ALM	III	C/SQ	D594N	G13D	Ma-Mel-30	D594N	G13D	13.6
34	46	f	skin	ALM	IV	LN	wt	wt	Ma-Mel-31	wt	Q61R	55.1
35	14	f	skin	n.a.	IV	LN	V600E	wt	Ma-Mel-32	V600E	wt	0.8
36	62	m	skin	NM	IV	C/SQ	V600E	wt	Ma-Mel-33	V600E	wt	11.2
37	72	f	skin	amelanotic	IV	C/SQ	wt	Q61R	Ma-Mel-35	wt	Q61R	53.5+
38	27	f	skin	n.a.	IV	C/SQ	V600E	wt	Ma-Mel-36	V600E	wt	4.0
39	59	f	skin	NM	III	LN	wt	wt	Ma-Mel-37b	wt	Q61L	63.2+
40	63	f	skin	n.a.	IV	LN	V600E	wt	Ma-Mel-38	V600E	wt	7.0
41	57	f	skin	SSM	IV	C/SQ	wt	wt	Ma-Mel-39a	wt	wt	12.6
42	61	m	skin	SSM	IV	C/SQ	n.d.	n.d.	Ma-Mel-40	wt	wt	10.4
43	37	f	skin	SSM	IV	C/SQ	V600E	wt	Ma-Mel-41	V600E	wt	12.7
44	80	f	occult	occult	IV	ascites	n.d.	n.d.	Ma-Mel-42	wt	wt	0.7
45	48	f	skin	NM	IV	C/SQ	wt	Q61R	Ma-Mel-43	wt	Q61R	6.2
46	39	m	occult	occult	IV	brain	V600E	wt	Ma-Mel-45a	V600E	wt	4.3
47	51	f	skin	n.a.	IV	C/SQ	V600E	wt	Ma-Mel-46	V600E	wt	2.0
48	33	f	skin	NM	III	LN	V600E	wt	Ma-Mel-47	V600E	wt	6.2
49	87	m	skin	NM	IV	C/SQ	G469R	wt	Ma-Mel-48a	G469R	wt	13.8
50	71	m	occult	occult	IV	ascites	n.d.	n.d.	Ma-Mel-49	wt	wt	1.7
51	62	m	skin	SSM	IV	ascites	n.d.	n.d.	Ma-Mel-50	wt	wt	1.6
52	48	m	occult	occult	IV	LN	V600E	wt	Ma-Mel-51	V600E	wt	2.0
53	62	m	skin	SSM	IV	C/SQ	V600K	wt	Ma-Mel-52	V600K	wt	0.5
54	69	m	skin	SSM	IV	C/SQ	wt	Q61K	Ma-Mel-53	wt	Q61K, R68T	43.3+
55	41	f	skin	SSM	IV	LN	V600E	wt	Ma-Mel-54a	V600E	wt	3.7
56	39	m	skin	NM	IV	LN	V600E	wt	Ma-Mel-55	V600E	wt	8.1
57	65	f	skin	SSM	III	LN	wt	Q61K	Ma-Mel-56	wt	Q61K	0.6
58	78	m	skin	n.a.	IV	LN	V600E	wt	Ma-Mel-57	V600E	wt	3.8
59	54	f	skin	SSM	IV	ascites	n.d.	n.d.	Ma-Mel-58	wt	wt	1.8
60	72	m	skin	NM	IV	LN	V600E	wt	Ma-Mel-59a	V600E	wt	2.6
61	58	m	skin	NM	IV	C/SQ	wt	Q61K	Ma-Mel-60	wt	Q61K	7.4
62	29	m	occult	occult	IV	LN	V600E	wt	Ma-Mel-61	V600E	wt	31.3+
63	17	m	skin	NM	IV	LN	wt	wt	Ma-Mel-62	wt	wt	4.2
64	44	f	occult	occult	IV	C/SQ	n.d.	n.d.	Ma-Mel-63a	V600E	wt	2.7
65	59	m	occult	occult	IV	ascites	n.d.	n.d.	Ma-Mel-64	wt	wt	0.6
66	24	f	skin	SSM	III	LN	wt	Q61K	Ma-Mel-65	wt	Q61K	26.1+
67	66	f	skin	NM	IV	C/SQ	V600E	wt	Ma-Mel-66a	V600E	wt	0.8
68	51	f	skin	SSM	IV	LN	V600K	wt	Ma-Mel-67	V600K	wt	26.7+
69	43	f	skin	NM	III	LN	wt	Q61R	Ma-Mel-68	wt	Q61R	6.0
70	61	m	skin	NM	IV	pleural effusion	n.d.	n.d.	Ma-Mel-69	V600K	wt	2.2
71	76	f	skin	SSM	IV	C/SQ	wt	wt	Ma-Mel-70	wt	wt	11.9
72	46	m	skin	ALM	IV	C/SQ	wt	wt	Ma-Mel-71	wt	wt	4.0
73	69	m	skin	n.a.	IV	LN	wt	Q61H	n.a.	n.d.	n.d.	25.7+
74	75	m	skin	ALM	III	LN	wt	wt	Ma-Mel-73b	wt	wt	7.5
75	59	f	skin	NM	III	LN	wt	Q61R	Ma-Mel-74	wt	Q61R	20.3+
76	60	m	skin	ALM	III	LN	wt	wt	n.a.	n.d.	n.d.	12.0
77	63	f	mucosa	mucosal	III	LN	wt	wt	Ma-Mel-76	wt	wt	5.6
78	41	m	skin	NM	IV	C/SQ	wt	wt	Ma-Mel-79b	wt	Q61K	10.6+
79	63	f	skin	NM	III	LN	V600E	wt	Ma-Mel-80a	V600E	wt	9.3+
80	30	f	skin	n.a.	IV	C/SQ	wt	wt	Ma-Mel-81	wt	wt	2.1
81	65	m	skin	SSM	IV	LN	wt	G12D	Ma-Mel-82	wt	G12D	12.6
82	42	f	skin	n.a.	IV	LN	V600E	wt	Ma-Mel-83	V600E	wt	7.0
83	38	m	occult	occult	IV	C/SQ	V600E	wt	Ma-Mel-85	V600E	wt	3.3
84	34	f	skin	NM	III	LN	V600E	wt	Ma-Mel-86a	V600E	wt	38.5+
85	45	m	skin	n.a.	IV	C/SQ	wt	wt	Ma-Mel-90	wt	wt	4.5
86	69	m	skin	NM	III	C/SQ	wt	Q61K	Ma-Mel-91	wt	Q61K	19.7
87	63	f	skin	n.a.	III	C/SQ	V600E	wt	Ma-Mel-92d	V600E	wt	11.3
88	75	m	occult	occult	IV	C/SQ	wt	G13R	Ma-Mel-93	wt	G13R	9.2
89	65	m	skin	NM	III	LN	wt	Q61R	Ma-Mel-94	wt	Q61R	3.9
90	45	m	skin	n.a.	IV	C/SQ	V600E	wt	Ma-Mel-96	V600E	wt	6.2
91	38	f	skin	SSM	III	LN	V600E	wt	Ma-Mel-97	V600E	wt	13.0+
92	32	m	skin	NM	III	C/SQ	wt	wt	n.a.	n.d.	n.d.	8.2+
93	59	m	n.a.	n.a.	IV	C/SQ	V600K	wt	Ma-Mel-99	V600K	wt	3.4
94	83	m	skin	NM	III	LN	wt	Q61K	Ma-Mel-100a	wt	Q61K	12.9+
95	34	f	skin	n.a.	IV	C/SQ	V600E	wt	Ma-Mel-101	V600E	wt	2.0
96	74	m	skin	SSM	III	LN	wt	Q61L	Ma-Mel-102a	wt	Q61L	10.4+
97	48	f	skin	NM	IV	C/SQ	wt	wt	Ma-Mel-103b	wt	wt	4.8
98	43	f	skin	NM	IV	lung	V600E	wt	Ma-Mel-104	wt	wt	10.0+
99	48	f	occult	occult	IV	liver	wt	Q61R	Ma-Mel-105	wt	Q61R	7.2+
100	76	m	skin	ALM	III	LN	wt	wt	Ma-Mel-107	wt	wt	5.4
101	35	f	occult	occult	IV	C/SQ	V600E	wt	Ma-Mel-108	V600E	wt	8.8
102	75	m	skin	LMM	III	LN	wt	wt	Ma-Mel-112	wt	wt	5.4+
103	60	m	skin	SSM	III	LN	V600E	wt	Ma-Mel-113	wt	wt	7.0+
104	67	m	skin	n.a.	IV	brain	wt	wt	Ma-Mel-114	wt	wt	6.6
105	74	m	skin	SSM	IV	C/SQ	wt	wt	Ma-Mel-119	wt	wt	4.2+
106	63	m	skin	n.a.	III	LN	V600E	wt	Ma-Mel-120	V600E	wt	10.0+
107	20	m	skin	NM	III	LN	V600E	wt	Ma-Mel-121a	wt	wt	7.0+
108	47	m	occult	occult	III	LN	V600E	wt	Ma-Mel-122	V600E	wt	4.3+
109	67	f	mucosa	mucosal	IV	C/SQ	wt	wt	Ma-Mel-123	wt	wt	6.3+

DNA was extracted from 97 tissue biopsies and 105 biopsy-derived cell lines from 109 metastatic melanoma patients, and screened for mutations in exons 11 and 15 of the B-RAF gene and exons 1 and 2 of the N-RAS gene. AJCC, American Joint Committee on Cancer; NM, nodular melanoma; SSM, superficial spreading melanoma; ALM, acrolentiginous melanoma; LMM, lentigo maligna melanoma; occult, melanoma of unknown primary; C/SQ, cutaneous or subcutaneous metastasis; LN, lymph node metastasis; wt, wildtype; n.a., not available; n.d., not done.

### Mutations in B-RAF and N-RAS genes

Screening of exons 11 and 15 of the B-RAF gene resulted in the detection of mutations in 53/97 (54.6%) tissue biopsies and 53/105 (50.5%) biopsy-derived cell lines (***Tables 2*** ***and*** ***3***). The most common mutation in the B-RAF gene was T1799A detected in 46/105 (43.7%) cell lines and 47/97 (48.5%) tissue biopsies. This mutation causes a change from valine to glutamic acid at codon 600 in exon 15 (V600E). Five cell lines carried the GT1798-99AA mutation at codon 600 (V600K). The only non-600 codon mutation in exon 15 was G1780A (D594N), found in one single cell line (Ma-Mel-30). All of the B-RAF mutations in exon 15 were concordant in cell lines and corresponding tissues except in five cases: The cell line UKRV-Mel-29 but not the corresponding tissue sample carried the V600K mutation, whereas the corresponding tissue biopsies but not the cell lines UKRV-Mel-11, Ma-Mel-104, Ma-Mel-113 and Ma-Mel-121a carried the V600E mutation. In exon 11 of the B-RAF gene we detected two mutations, G469R and G469V. The first was found in the cell line Ma-Mel-48a and its corresponding tissue, whereas the latter was present in a tissue biopsy with no corresponding cell line available.

**Table 3:** Overview on B-RAF and N-RAS mutation status.

	Tissues	Cell lines
	97 (100.0%)	105 (100.0%)
**B-RAF mutation**	**53 (54.6%)**	**53 (50.5%)**
*** Exon 11***	***2 (2.0%)***	***1 (1.0%)***
G469R	1 (1.0%)	1 (1.0%)
G469V	1 (1.0%)	0 (0.0%)
*** Exon 15***	***51 (52.6%)***	***52 (49.5%)***
D594N	1 (1.0%)	1 (1.0%)
V600E	47 (48.5%)	46 (43.7%)
V600K	3 (3.1%)	5 (4.8%)
**N-RAS mutation**	**22 (22.7%)**	**26 (24.8%)**
*** Exon 1***	***3 (3.1%)***	***4 (3.8%)***
G12D	1 (1.0%)	2 (1.8%)
G13D	1 (1.0%)	1 (1.0%)
G13R	1 (1.0%)	1 (1.0%)
*** Exon 2***	***19 (19.6%)***	***22 (21.0%)***
Q61R	11 (11.3%)	12 (11.4%)
Q61L	1 (.0%)	2 (1.8%)
Q61K	6 (6.3%)	8 (7.8%)
Q61H	1 (1.0%)	0 (0.0%)
R68T	0 (.0%)	1* (1.0%)
**B-RAF ** ***or*** ** N-RAS mutation**	**73 (75.3%)**	**77 (73.3%)**
**B-RAF ** ***and*** ** N-RAS mutation**	**1 (1.0%)**	**1 (1.0%)**
***No*** ** B-RAF or N-RAS mutation**	**23 (23.7%)**	**27 (25.7%)**

Tumor tissue biopsies and biopsy-derived cell lines from 109 metastatic melanoma patients were analysed for B-RAF and N-RAS mutations. For details see *Figure 1* and *Table 2*. *This cell line additionally carries the N-RAS Q61K mutation.

Mutations in the N-RAS gene mostly occurred at codon 61 of exon 2 and were present in 19/97 (19.6%) tissue biopsies and 22/105 (21.0%) biopsy-derived cell lines (***Tables 2*** ***and*** ***3***). The cell line Ma-Mel-53 and its corresponding tissue in addition to the codon 61 mutation carried a second N-RAS mutation at codon 68 (R68T). Four cell lines (Ma-Mel-31, Ma-Mel-37b, Ma-Mel-79b and Ma-Mel-53) revealed mutations in exon 2 (Q61R, Q61L, Q61K and R68T, respectively) that could not be detected in the corresponding tissue biopsies. One mutation (Q61R) detected in a tumor tissue was not present in the corresponding cell line Ma-Mel-02. Mutations in exon 1 at codon 12 and 13 of the N-RAS gene were detected in four cell lines and three corresponding tissue samples, respectively. The mutation G12D in the cell line Ma-Mel-27 could not be detected in the matching tissue biopsy.

Taken together, 73/97 (75.3%) tumor tissue biopsies and 77/105 (73.3%) biopsy-derived cell lines carried mutually exclusive mutations in B-RAF or N-RAS (***Table 3***). Only one cell line (Ma-Mel-30) and corresponding tissue carried mutations in both, B-RAF (D594N) and N-RAS (G13D) genes.

### Differential impact of B-RAF and N-RAS mutations on survival

**Figure 3:**
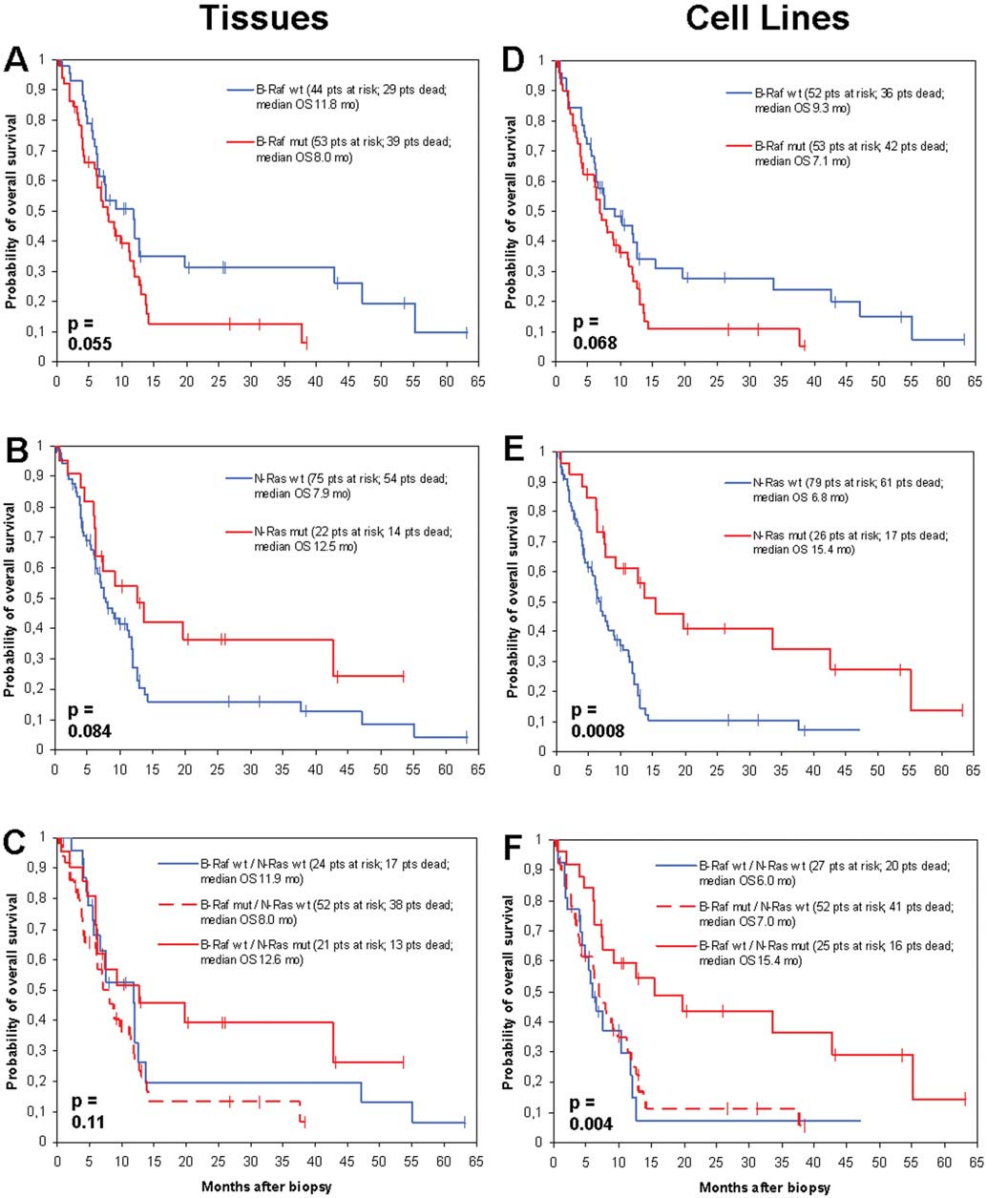
Kaplan-Meier survival estimation for the whole patient population by mutational status. Curves showing the overall survival of 109 metastatic melanoma patients starting from the time point of tumor biopsy. Survival probabilities were compared by the mutational status of B-RAF in tumor tissue biopsies (n = 97) (A) and biopsy-derived tumor cell lines (n = 105) (D), as well as N-RAS in tumor tissue biopsies (n = 97) (B) and biopsy-derived tumor cell lines (n = 105) (E). (C) and (F) differentiate patients harbouring B-RAF mutations (n = 52, tissues; n = 52, cell lines), patients harbouring N-RAS mutations (n = 21, tissues; n = 25; cell lines), and patients without mutations in both genes (n = 24, tissues; n = 27, cell lines). Statistical differences between groups were calculated using the log-rank test. Vertical bars indicate censored observations.

**Figure 4:**
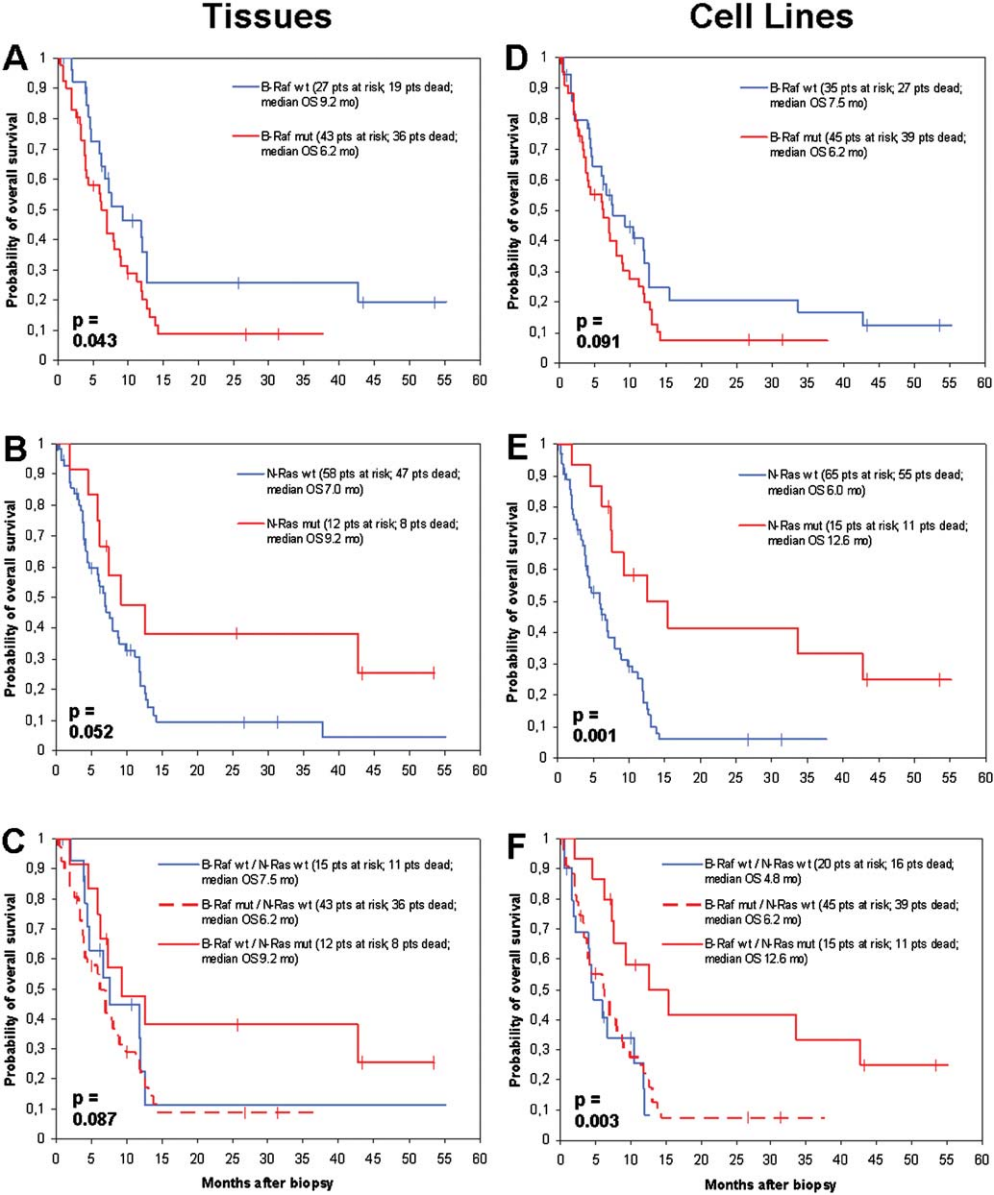
Kaplan-Meier survival estimation for stage IV patients only by mutational status. Curves showing the overall survival starting with the time point of tumor biopsy in 82 metastatic melanoma patients who were in stage IV disease at that time. Survival probabilities were compared by the mutational status of B-RAF in tumor tissue biopsies (n = 70) (A) and biopsy-derived tumor cell lines (n = 80) (D), as well as N-RAS in tumor tissue biopsies (n = 70) (B) and biopsy-derived tumor cell lines (n = 80) (E). (C) and (F) differentiate patients harbouring B-RAF mutations (n = 43, tissues; n = 45, cell lines), patients harbouring N-RAS mutations (n = 12, tissues; n = 15; cell lines), and patients without mutations in both genes (n = 15, tissues; n = 20, cell lines). Statistical differences between groups were calculated using the log-rank test. Vertical bars indicate censored observations.

During a median follow-up time of 31.0 months, 80 (73.4%) out of 109 patients died from melanoma. Patients whose tumor tissue biopsies revealed a mutation in the B-RAF gene showed a decreased probability of overall survival from date of biopsy compared to patients without a B-RAF mutation (median 8.0 versus 11.8 months, p = 0.055; ***Figure 3A***). This correlation of borderline significance could similarly be detected in patients, from whose tumor biopsies permanently growing cell lines could be established (B-RAF mutation compared to wildtype, median overall survival 7.1 versus 9.3 months, p = 0.068; ***Figure 3D***). Patients carrying an N-RAS mutation in their tumor tissue biopsies revealed an improved survival compared to patients without an N-RAS mutation (median 12.5 versus 7.9 months, p = 0.084; ***Figure 3B***). This association could be detected to a stronger extent in patients whose biopsy-derived tumor cell lines carried an N-RAS mutation compared to those patients without such mutation (median overall survival 15.4 versus 6.8 months, p = 0.0008; ***Figure 3E***). Looking at a subgroup of 82 patients who were in stage IV at the time of tumor biopsy, again patients harbouring a B-RAF mutation showed a reduced overall survival compared to patients with wildtype B-RAF (p = 0.043, tissues, ***Figure 4A***; p = 0.091, cell lines, ***Figure 4D***), whereas patients holding an N-RAS mutation presented a favorable survival compared to patients without N-RAS mutation (p = 0.052, tissues, ***Figure 4B***; p = 0.001, cell lines, ***Figure 4E***).

With regard to overall survival measured from first diagnosis of melanoma, no significant differences were seen by B-RAF or N-RAS mutation status, respectively, neither for patients whose mutation status was determined from tissue specimens, nor for those, whose mutation status was measured in cell lines (data not shown). Calculating overall survival starting with the first diagnosis of metastasis, patients carrying a B-RAF mutation revealed a trend to a decreased survival compared to patients with wildtype B-RAF (p = 0.052, tissues; p = 0.072, cell lines), whereas patients carrying an N-RAS mutation showed a trend towards a favorable survival compared to patients without N-RAS mutation (p = 0.18, tissues; p = 0.12, cell lines).

A multivariate analysis using the proportional hazards model of Cox revealed the disease stage at biopsy as the only factor of independent prognostic impact on overall survival from date of biopsy with regard to tissue analysis (p = 0.03; ***Table 4***). B-RAF and N-RAS mutation status both showed a p = 0.19). Site of primary (p = 0.27), and gender (p = 0.79) did not show major influence on survival. The analysis of biopsy-derived tumor cell lines revealed the N-RAS mutation status as the strongest prognostic factor (p = 0.006), followed by disease stage at biopsy (p = 0.02), site of primary (p = 0.14), and B-RAF mutation status (p = 0.29). Similar data were obtained analysing the subgroup of 82 stage IV patients (***Table 4***). Additional analyses were performed dividing the patients into three groups, (i) patients harbouring B-RAF mutations, (ii) patients harbouring N-RAS mutations, and (iii) patients without mutations in both genes. These analyses revealed, that with regard to the entire patient population (n = 109), patients harbouring B-RAF mutations show a similar survival as patients without a mutation in B-RAF or N-RAS, whereas patients carrying an N-RAS mutation present with a favorable survival (p = 0.11, tissues, ***Figure 3C***; p = 0.004, cell lines, ***Figure 3F***). This finding could be similarly observed when looking at the subgroup of 82 patients, whose tumor biopsy was obtained during stage IV disease (p = 0.087, tissues, ***Figure 4C***; p = 0.003, cell lines, ***Figure 4F***).

**Table 4:** Multivariate survival analysis.

	all patients			stage IV patients		
Variable	Hazard ratio	95% CI	***P***	Hazard ratio	95% CI	***P***
**Tissue biopsies**						
* gender*						
male	1	0.4 to 1.9	0.79	1	0.5 to 1.7	0.87
female	0.9			1.0		
* site of primary*						
skin	1	0.5 to 2.2	0.27	1	0.4 to 2.4	0.99
other	1.1			1.0		
* stage at biopsy*						
III	1	1.3 to 4.9	***0.03***	n.a.		
IV	2.0					
* B-RAF*						
wt	1	0.8 to 3.1	0.19	1	0.6 to 3.0	0.41
mutation	1.4			1.4		
* N-RAS*						
wt	1	0.3 to 1.1	0.19	1	0.2 to 1.7	0.34
mutation	0.6			0.6		
**Biopsy-derived cell lines**						
* gender*						
male	1	0.4 to 2.0	0.75	1	0.6 to 1.8	0.91
female	0.9			1.0		
* site of primary*						
skin	1	0.6 to 2.5	0.14	1	0.7 to 2.9	0.30
other	1.2			1.4		
* stage at biopsy*						
III	1	1.4 to 4.2	***0.02***	n.a.		
IV	2.3					
* B-RAF*						
wt	1	0.8 to 3.0	0.29	1	0.7 to 3.3	0.36
mutation	1.3			1.4		
* N-RAS*						
wt	1	0.1 to 0.8	***0.006***	1	0.1 to 0.6	***0.002***
mutation	0.4			0.3		

The prognostic impact of multiple variables was analysed using the multivariate proportional hazards regression of Cox. Overall survival was calculated beginning with the date of tumor biopsy. wt, wildtype; CI, confidence interval; n.a., not applicable.

## Discussion

The strong concordance between the mutational status of tissues and corresponding cell lines, showing a differing status for B-RAF in only 5% and N-RAS in only 6%, respectively, strongly argues against the notion that in vitro short term propagation biases the frequency of mutational events in genes encoding the MAPK signaling pathway. The finding that these mutations are preserved throughout in vitro tumor propagation suggests, that they may influence tumor maintenance. In support of this idea, studies in a mouse model system have shown, that activated RAS is required for melanoma maintenance [14]. Moreover, our findings validate earlier reports using short term propagated melanoma cell culture for genotypic analysis.

Until recently, it has been anticipated that B-RAF and N-RAS mutations result in an activation of the RAS/RAF/MEK/ERK signalling pathway, thus in a comparable cellular phenotype which would similarly influence the clinical outcome of melanoma patients. Consequently, the majority of previous reports considered patients with mutations in B-RAF *or* N-RAS as one prognostic group. In this regard, Houben et al. showed mutations in B-RAF *or* N-RAS to be associated with an impaired overall survival in patients with metastatic melanoma [5]. Similarly, Daniotti et al. studied patient-derived melanoma cell lines and found a correlation of mutations in either B-RAF *or* N-RAS with poor overall survival [8]. However, Dumaz et al. demonstrated that cAMP suppresses C-RAF activity in melanocytes, and that this suppression is essential to decrease the oncogenic potential of C-RAF in these cells [15]. As a result, B-RAF alone is responsible for signaling to MEK. When N-RAS is mutated, though, cells switch their signalling from B-RAF to C-RAF, i.e. a fundamental switch in RAF isoform usage occurs when RAS is mutated in melanoma. Indeed, in a recent study comparing gene expression profiles of melanoma cell lines with either mutations in B-RAF or N-RAS, we found twice as many upregulated genes in cell lines carrying N-RAS mutations than in those carrying mutations in B-RAF, with an overlap of only 16% [16]. Pathway analysis of the affected genes suggested, that the major B-RAF mutation V600E mainly affects the ERK signaling pathway, whereas mutations in N-RAS cause perturbation of the expression in genes involved in the PI3K/AKT apoptotic pathway.

These observations should have important implications for the analysis of the prognostic relevance of B-RAF and N-RAS mutations as well as the development of therapeutic strategies to treat this life-threatening disease [17]. Many of the newly developed targeted therapeutics are multikinase inhibitors, but nevertheless exert affinities of different strength to different kinases. Sorafenib (BAY 43-9006), a multikinase inhibitor recently tested in metastatic melanoma with significant efficacy, has a much stronger affinity to RAF compared with that to RAS, whereas farnesyl transferase inhibitors interfere with the translocation of RAS but not RAF to the cell membrane. With regard to this issue, we distinguished between B-RAF and N-RAS mutations in regard to their influence on survival of metastatic melanoma patients. Surprisingly, this analysis revealed that patients carrying B-RAF mutations had an impaired survival, whereas patients with N-RAS mutations were characterized by a favorable prognosis, each in comparison with the wildtype gene status. Moreover, multivariate data analysis showed that N-RAS but not B-RAF mutation status was an independent prognostic factor.

The mechanisms how N-RAS mutations contribute to an improved survival of melanoma is not yet fully understood. However, it could be speculated to be related to the differences in the downstream effectors between RAS and RAF. As mentioned above, it has been presumed until recently, that the primary function of RAS was simply to facilitate RAF activation. However, the discovery of other proteins that are effectors of RAS function suggested, that oncogenic activities of RAS are mediated by both RAF-dependent and RAF-independent signalling. Notably, further complexity arose with the identification of RAS effectors (e.g. RASSF1-2 or NORE1) with putative tumor suppressor, rather than oncogenic functions [18]. Our previous analysis of gene expression profiles of melanoma cell lines with either mutations in B-RAF or N-RAS revealed substantial differences including expression of tumor suppressor genes and oncogenes [16]. In this respect, it is important to note, that Demunter et al. described an N-RAS mutation at codon 18 in melanoma tissues, that was associated with a favorable disease outcome [19]. However, this particular mutation was not detected in the tumor material analyzed in our present study. Nevertheless, the association of oncogenic mutations with a favorable prognosis is not without a precedent. In bladder cancer, FGFR3 mutations have been associated with a prolonged survival and tumors carrying these mutations constitute a favorable disease category [20]. A completely different explanation may rely on the different immunogenicity of mutated B-RAF and N-RAS or the respective induced effector molecules, as it has been recently suggested for the seemingly paradoxical association of a bcl-2 over-expression with an improved prognosis in cancer patients [21].

The negative impact of B-RAF mutations on survival was slightly more apparent in the analysis of tissues than in the analysis of cell lines. In contrast, N-RAS mutations were associated with a favorable prognosis, though statistical significance was reached only in results from cell lines. One of the possible reasons might be that we did not succeed in establishing a cell line from every tissue biopsy, resulting in an unintended selection bias, which might favor the establishment of cell lines carrying N-RAS mutations. This is supported by our observation that in five cases mutations in the N-RAS gene were present in tumor cell lines but not in the corresponding tissue samples. Another unbalanced parameter was based on the inclusion of malignant effusions, from which only cell lines but no corresponding tissues could be derived. This fact might have created a bias between the patient populations from whom tumor cell lines and/or tissues could be analysed, which is of particular relevance because melanoma patients presenting malignant effusions are known to show an extremely poor prognosis. Our study population included seven cell lines derived from malignant effusions, all of which were negative for N-RAS mutations. This observation might explain the differences seen between N-RAS mutated and wildtype cell lines towards prognosis.

Taken together, we demonstrate that the mutational status of B-RAF and N-RAS are well preserved during short term in vitro propagation, and, most importantly, that B-RAF and N-RAS mutations differentially impact the outcome of melanoma patients. These findings should be considered in conjunction with therapeutic strategies under current investigation, using B-RAF and N-RAS as molecular targets [22], e.g. considering the observation that inhibitors of N-RAS like farnesyltransferase inhibitors might not be effective in melanoma therapy [23]. Future clinical trials in this field should be accompanied by a focused molecular workup of patient material in order to provide further insights into the impact of mutational profiles on the prognosis and therapy response of melanoma patients.
